# The educational pathway to Advanced Practice for the physiotherapist: Protocol for a systematic mixed studies review

**DOI:** 10.1371/journal.pone.0308921

**Published:** 2024-09-27

**Authors:** Kaitlyn Maddigan, Katie L. Kowalski, Andrews K. Tawiah, Alison B. Rushton

**Affiliations:** School of Physical Therapy, Faculty of Health Sciences, Western University, London, Ontario, Canada; University of the Witwatersrand Johannesburg, SOUTH AFRICA

## Abstract

**Rationale:**

Advanced Practice Physiotherapy (APP) is a post-licensure higher level of practice that requires distinctly increased skills, clinical reasoning and experience. The four pillars that underpin APP are clinical practice, leadership, education and research. Multiple systematic reviews support that APP is beneficial to health care systems. While APP exists in over a dozen countries, it has yet to reach international recognition. A steppingstone in gaining global acknowledgement is understanding the educational pathway that physiotherapists traverse to become Advanced Practitioners. No systematic review has synthesized evidence to describe and evaluate the educational pathway for physiotherapists to APP. Therefore, the objectives of this review are 1) to describe the post-licensure educational pathways that physiotherapists engage in to advance their level of practice, 2) to evaluate the pillars of APP demonstrated by the physiotherapist after traversing a post-licensure educational pathway.

**Materials and methods:**

A systematic mixed studies review using a data based convergent qualitative synthesis design will be conducted. MEDLINE (Ovid), Embase, CINAHL, the Cochrane Library, Web of Science, PEDro, SportDiscus, ProQuest Education databases as well as the grey literature will be searched from inception to 02/29/2024. Studies that aim to describe and or evaluate the capacity of educational pathways to influence the level of practice of the physiotherapist will be included. Two independent reviewers will screen studies, extract data and assess methodological quality (Quality Assessment of Diverse Studies). Quantitative data will be ‘qualitized’, and all data will be synthesized via a clustered textual description and directed content analysis. After synthesis, two reviewers will assess confidence in the cumulative evidence (GRADE-CERQual), which will inform the discussion.

**Implications:**

The optimal pathway(s) to Advanced Practice for the physiotherapist will be evaluated to inform future high-quality research investigating the effectiveness of post-licensure education in developing Advanced Practice physiotherapists.

## Introduction

### Rationale

Traditional health care systems are typically structured around the needs and capabilities of specific disciplines, with physicians being the main decision-makers for referrals, admissions, and discharges [1]. This confined route to accessing care, has led to long and disordered experiences for patients without immediate life-threatening conditions [2–4]. The repercussions include one in three people globally living with conditions that would benefit from rehabilitation services [5, 6]. Adding to this problem, is a shortage of primary care providers; the World Health Organization reports that half of the global population lacks access to essential health services [7]. This situation is exacerbated in low- and middle-income countries [8, 9] but it is not unique to them [10–12], for example, one in five Canadians do not have a general practitioner [13]. Consequently, patients resort to the emergency department for their healthcare concerns–an overcrowded, similarly constrained path, that poses long wait times, high costs to the system and disintegrated care [6, 10–12, 14–16]. An emerging solution to this global dilemma is an interprofessional model that uses the skill set of Advanced Practice physiotherapists at the primary, secondary or tertiary levels of care [17, 18]. Several systematic reviews have supported APP as a solution to burdened health care systems; particularly in the field of musculoskeletal disorders, rheumatology and the emergency department. These reviews indicate that Advanced Practice physiotherapists provide accurate diagnosis, triage appropriately, and generally improve both access to care and treatment outcomes for a range of patients [19–25].

APP began in the 1970s, out of the United States military to address the lack of physician capacity to attend to injured soldiers, since then it has evolved to span fifteen fields of practice, in fourteen countries and territories around the world [18]. Currently, there is no agreed upon protected title to recognize APP internationally, however according to the 2019 World Physiotherapy policy statement, there is consensus on what APP is. It is a higher level of practice that requires distinctly increased skills, clinical reasoning and experience, that leads to improved service outcomes, patient experiences and involves providing care to patients with complex needs, both safely and competently [26, 27]. Moreover, it is widely accepted in the United Kingdom that APP is underpinned by the four pillars of clinical practice, education (facilitating learning), leadership (management) and research development [27].

A current gap in the evolution of APP internationally is the lack of a globally defined education pathway–historically, education has involved on the job training, with no specified standards or regulations to be met [18]. However, World Physiotherapy has recently issued a call to action for the provision of appropriate educational frameworks [26]. Themes of what those frameworks might entail are emerging from around the world, but the landscape is incomplete. In the UK, a trailblazing country in the legislation of APP [28], the educational pathway to Advanced Practice is typically a post-graduate (post-licensure) Masters level qualification, although an alternative portfolio-based route is recognized as an equivalent mode of development, provided that the practitioner can demonstrate evidence of the competencies of all four pillars [27]. Additionally, in a recent survey of one hundred and twelve member organizations of World Physiotherapy, half of the respondents agreed that Advanced Practice physiotherapists should demonstrate a set of defined competencies to be considered for an APP role and that clinicians in these roles must have a post-graduate qualification such as a Masters degree or PhD [18].

There is agreement among stakeholders regarding the definition of APP and its potential to provide relief to many sectors of the healthcare system. However, discord remains on the international stage when it comes to an established educational pathway to APP [29]. Lessons learned from the nursing profession highlight the importance of standardization of the educational pathway to Advanced Practice, recognizing that growth, acknowledgement and integration of Advanced Practice roles has previously been supported after achieving a milestone of this nature [30]. Furthermore, a standardized educational pathway to APP can help to standardize education of practitioners, increase transferability of skills and ultimately improve the quality of patient care given the provision of high-quality education [28, 29]. It is therefore a priority to undertake a review of the literature to describe and evaluate the educational pathways to APP internationally, to understand what is known already about the physiotherapist’s educational journey to achieving the required competencies across the four pillars of Advanced Practice and to identify any gaps in knowledge that would call for further research.

### Objectives

1. To describe the post-licensure educational pathways that physiotherapists engage in to advance their level of practice.
2. To evaluate the pillars of Advanced Practice demonstrated by the physiotherapist after traversing a post-licensure educational pathway.

## Materials and methods

This protocol is reported in line with the Preferred Reporting Items for Systematic review and Meta-Analysis Protocols (PRISMA-P) (see S1 Table) [31] and is registered with the international prospective register of systematic reviews, PROSPERO CRD42024499563.

### Researcher positionality

All authors are registered physiotherapists working in post-secondary academia in Ontario, Canada with a focus on research and education in APP. The primary author is female, with a professional journey rooted in the fields of high-performance sport and musculoskeletal physiotherapy which has included the completion of a Master of Clinical Science in Advanced Health Care Practice, as well as earning the designation Registered International Sport Physical Therapist and Fellow of the Canadian Academy of Manipulative Physiotherapists. In addition to being actively engaged in shaping the next generation of Advanced Practitioners as a university educator, she currently holds a clinical Advanced Practice position in Ontario, Canada. This multifaceted role is at the intersection of theory and application, it provides the opportunity to bridge clinical expertise with academic responsibilities and offers a unique vantage point on the educational pathways to APP. Finally, the first author is studying towards a PhD in Health and Rehabilitation Sciences.

Epistemologically, the first author navigates between a post-positivist and a pragmatic paradigm. Recognising the importance of empirical evidence while simultaneously acknowledging the inherent complexities of the healthcare system, she approaches this systematic review with a commitment to balance rigor and relevance. The exploration of educational pathways demands an appreciation for both the objective realities and the practical considerations shaping the professional landscape, as such a mixed studies approach was chosen for this review. Ultimately, her positionality reflects a commitment to unraveling the intricacies of educational frameworks for Advanced Practice physiotherapists, drawing from her own experiences, academic engagement, and a pragmatic lens that aligns with the ever-evolving demands of the healthcare sector.

### Design

A systematic mixed studies review (SMSR) will be conducted using a data based convergent synthesis design [32]. Specifically, all studies containing qualitative, quantitative or mixed data will be analyzed via the same qualitative synthesis method for each objective. This design will require data transformation prior to synthesis, as such any quantitative data will be thematically analyzed as described by Pluye and Hong [33]. A SMSR permits the combination of stories and numbers–each powerful in their own right–but together have the capacity to yield robust and highly practical understandings of complex interventions and programs. Therefore, given the complexity and intricacies of investigating the educational pathway to Advanced Practice for the physiotherapist, a SMSR, that utilizes a clustered textual description [34] to address objective one, and a directed content analysis, deduced by the pillars of Advanced Practice [26, 35], to address objective two–is the most suitable approach for the present study [33]. Moreover, systematic reviews are considered the gold-standard for evidence synthesis and thereby is the optimal approach here as opposed to a scoping review as this investigation aims to uncover international evidence, to produce statements to guide decision-making and policy development as well as identify and inform future areas of research [36].

### Eligibility criteria

Eligibility criteria (Table 1) were informed by the PICOS framework [37].

**Table 1:** Eligibility criteria.

Population	Physiotherapists (Physical Therapists)
**Intervention**	Post-licensure educational pathways of any form but most commonly Masters level or post-graduate education, mentorship programs, residency programs or workplace training across all clinical fields.
**Comparison**	N/A
**Outcomes**	1. description of post-licensure educational pathways 2. demonstration of the four Pillars of Advanced Practice by the physiotherapist
**Study Design**	All primary research studies (qualitative, quantitative and mixed methods)
**Publication Language**	English, or papers able to be sufficiently translated into English via Google Translate

### Information sources

MEDLINE (Ovid), Embase, CINAHL, the Cochrane Library, Web of Science, PEDro, SportDiscus and ProQuest Education databases will be searched from inception to 02/29/2024. Grey literature will be searched through ProQuest Dissertations and Theses, trial registers (ClinicalTrials.gov and World Health Organization International Clinical Trials Registry Platform) and Google (the first one hundred results will be screened for inclusion). Manual searching of the reference lists of included studies will identify any further articles that meet eligibility criteria. Expert researchers of APP will be consulted for identification of other potential studies. If studies cannot be retrieved, access requests will be made through contacting the authors by email.

### Search strategy

The search strategy was developed in collaboration with a Teaching and Learning Librarian at Western University. The search was constructed based on three concepts: the physiotherapist, post-licensure educational pathways and the competencies that underpin the four pillars of Advanced Practice [38]. The first two concepts were informed by initial scoping of the literature, expert opinion and previously published search strategies investigating similar ideas [39, 40]. The third concept was informed by the multi-professional framework for Advanced Clinical Practice in England [27] and wider research / policy documents intending to define the concept of Advanced Practice physiotherapy [26, 39–41].

The search strategy was initially developed in MEDLINE (Ovid) (Table 2) and will be adapted for other included databases (see S2 Table).

**Table 2:** MEDLINE (Ovid) search strategy.

**1**	**Physical Therapists/**
**2**	Physical Therapy Specialty/
**3**	exp Physical Therapy Modalities/
**4**	physiotherap*.tw,kf.
**5**	physio-therap*.tw,kf.
**6**	physical therap*.tw,kf.
**7**	1 or 2 or 3 or 4 or 5 or 6
**8**	Education, Graduate/
**9**	exp Education, Professional/
**10**	Education, Continuing/
**11**	Models, Educational/
**12**	Mentoring/
**13**	exp Inservice Training/
**14**	"Internship and Residency"/
**15**	Musculoskeletal Manipulations/
**16**	Problem-Based Learning/
**17**	Competency-Based Education/
**18**	"master* level".tw,kf.
**19**	"master* education".tw,kf.
**20**	"post-licens* adj3 education".tw,kf.
**21**	"educational intervention*".tw,kf.
**22**	"work experience".tw,kf.
**23**	"professional development".tw,kf.
**24**	"manual therapy".tw,kf.
**25**	"self-directed learning".tw,kf.
**26**	IFOMPT.tw,kf.
**27**	8 or 9 or 10 or 11 or 12 or 13 or 14 or 15 or 16 or 17 or 18 or 19 or 20 or 21 or 22 or 23 or 24 or 25 or 26
**28**	exp Professional Competence/
**29**	Clinical Competence/
**30**	exp Clinical Decision Making/
**31**	Clinical Reasoning/
**32**	exp Quality of Health Care/
**33**	"Delivery of Health Care"/
**34**	exp Patient Satisfaction/
**35**	Treatment Outcome/
**36**	"Attitude of Health Personnel"/
**37**	exp Professional Role/
**38**	exp Accreditation/
**39**	Career Mobility/
**40**	Leadership/
**41**	Learning/
**42**	Research/
**43**	Program Evaluation/
**44**	Patient-Centered Care/
**45**	Communication/
**46**	Evidence-Based Practice/
**47**	Risk Management/
**48**	Cooperative Behavior/
**49**	Mentors/
**50**	(clinic* adj3 expert*).tw,kf.
**51**	"clinic* practice".tw,kf.
**52**	(capabilit* adj3 "advanced practice").tw,kf.
**53**	((advanced or speciali* or consultant*) adj5 (practice or practitioner*)).tw,kf.
**54**	qualification.tw,kf.
**55**	((extended or expanded) adj3 (role* or scope*)).tw,kf.
**56**	"skill acquisition".tw,kf.
**57**	"knowledge translation".tw,kf.
**58**	"role model".tw,kf.
**59**	adaptable.tw,kf.
**60**	"self-directed learning".tw,kf.
**61**	innovat*.tw,kf.
**62**	"best practice".tw,kf.
**63**	Health Educators/
**64**	Patient Care Management/
**65**	Safety Management/
**66**	manag*.tw,kf.
**67**	"clinician scientist".tw,kf.
**68**	Research Personnel/
**69**	"best practice".tw,kf.
**70**	"job experience".tw,kf.
**71**	28 or 29 or 30 or 31 or 32 or 33 or 34 or 35 or 36 or 37 or 38 or 39 or 40 or 41 or 42 or 43 or 44 or 45 or 46 or 47 or 48 or 49 or 50 or 51 or 52 or 53 or 54 or 55 or 56 or 57 or 58 or 59 or 60 or 61 or 62 or 63 or 64 or 65 or 66 or 67 or 68 or 69 or 70
**72**	7 and 27 and 71

### Study records

#### Data management

Citations identified with the search strategy will be imported and stored in Covidence, a web-based software platform for systematic reviews. Duplicates will be automatically identified by the software and removed. After title and abstract screening, full texts will be uploaded and stored in Covidence. Eligibility screening at both the title/abstract and full-text stages will also occur in Covidence.

#### Selection process

**Fig 1:**
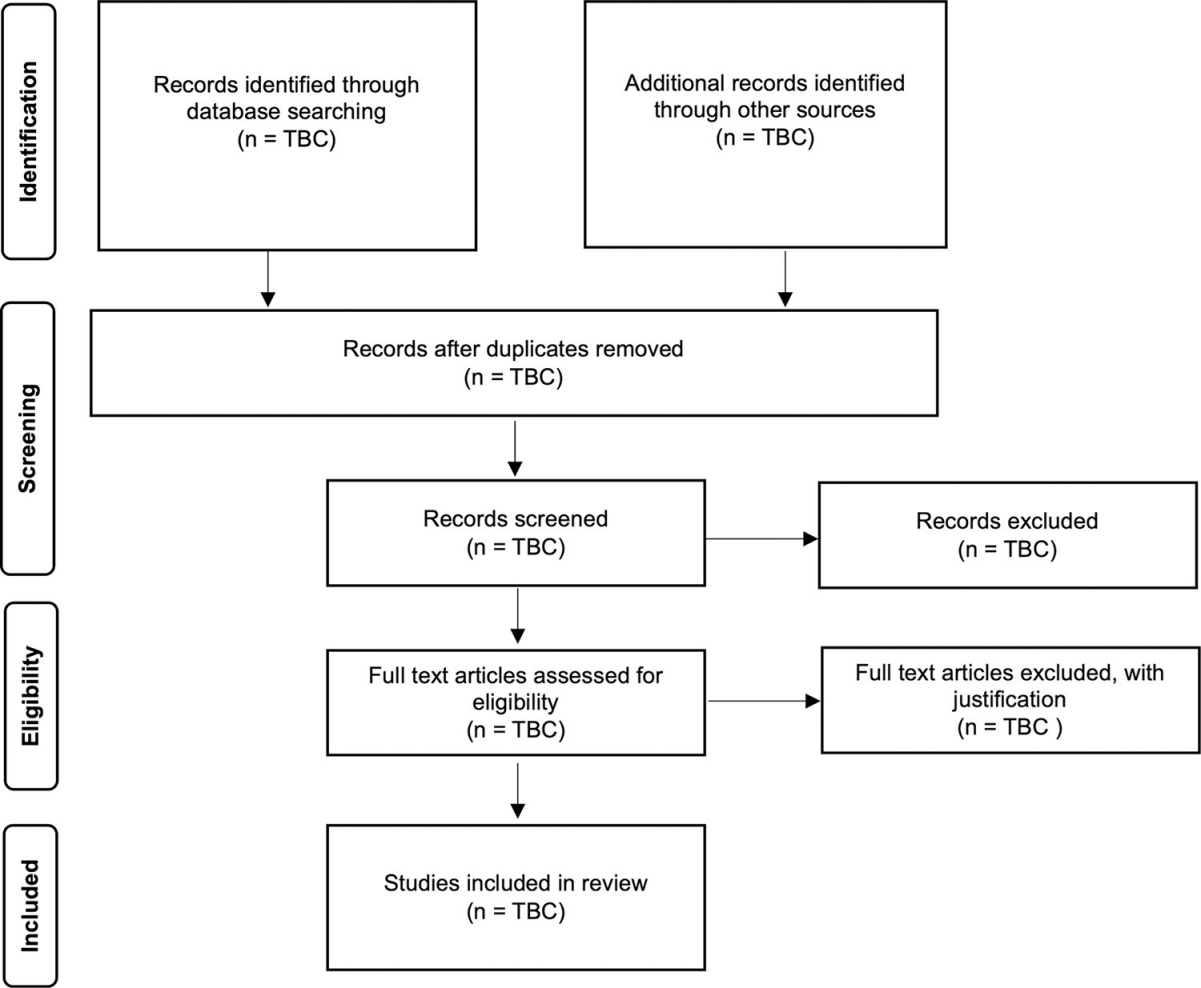
PRISMA flow diagram [42]. TBC = To be confirmed.

Two reviewers will independently determine eligibility. Pre-screening eligibility criteria training and instruction will be provided to reviewers before initiating the selection process. KM will perform searches and import citations into Covidence. Title and abstracts will be screened against eligibility criteria with full texts retrieved when both reviewers agree that eligibility criteria have been met. If the title and abstract does not include sufficient detail for determination of eligibility it will be marked ‘maybe’ and the full text will be obtained. Full text review will be completed independently by both reviewers. If there is agreement between both reviewers that the eligibility criteria have been met, the article will be included. At each stage, discrepancies will be discussed between reviewers and if consensus is not achieved, a third reviewer will mediate. Agreement between reviewers will be evaluated with Cohen’s kappa (k). Study identification, screening, eligibility, and inclusion will be presented using a PRISMA flow diagram (Fig 1) [31].

#### Data collection process

Two reviewers will independently extract data from eligible studies. Data will be extracted into a standardized form with pre-set data items. Discrepancies in data extracted will be resolved through discussion, a third reviewer will be included to mediate if needed.

#### Data items

Data items for extraction are detailed in Table 3.

**Table 3:** Data items.

author(s)
year of publication
country of data collection
study aim or objective
study design
study setting
sample size
method of data collection
characteristics of the educational pathway
characteristics of the physiotherapist (incl prior training and years of work experience)
demonstration of the competencies that underpin the four Pillars of Advanced Practice by the physiotherapist

### Outcomes and prioritization

The outcomes of interest in this review are the nature and characteristics of the post-licensure educational pathways that physiotherapists are engaging in to advance their level of practice as well as demonstration of the competencies that underpin the four pillars of Advanced Practice by the physiotherapist after traversing a post-licensure educational pathway [27].

### Quality assessment

The Quality Assessment for Diverse Studies (QuADS) will be used for methodological quality assessment of individual studies. The QuADS is a tool that was adapted from the Quality Assessment Tool for Studies with Diverse Designs for greater applicability to health services research [43]. The tool includes thirteen components, rated on a four-tiered rubric that aims to provide a basis for appraising the congruency, transparency and rigorous reporting of the research process for multiple-methods or mixed-methods study designs. The QuADS was selected for use over other mixed methods quality assessment tools due to it aligning with a post-positivistic paradigm, which aligns with the positionality of the primary author of this review. The QuADS tool demonstrates substantial inter-rater reliability (k = 0.66) [44], face and content validity for application in systematic reviews with mixed, or multi-methods health services research [43].

Two reviewers will pilot the QuADS before use to ensure agreement in scoring. They will then independently complete a methodological quality assessment of each included study. Evidence does not support that any criterion is more suggestive of quality than another or that a certain score signifies an overall high- or low-quality rating, therefore studies will not be excluded based on any cut-off score, as it would be arbitrary and unsupported by the literature [43]. Instead, the tool permits researchers to reflect and consider components of the study from a substantive position, gauging the extent to which each criterion is met. For this reason, the ratings of each criterion of the QuADS will be illustrated in a figure and accompanied by a descriptive report to better characterise the quality of included studies and inform the integrated synthesis and analysis of the extracted data.

### Data synthesis

SMSRs combine the power of stories and the power of numbers to yield robust and highly practical understandings of complex interventions and programs. One of the challenges encountered in these types of reviews is that of data synthesis owing to the heterogeneity of included study designs [33]. Hong et al (2017) advocate a data based convergent synthesis design in situations where, in line with this review’s objectives the SMSR seeks to identify and define main concepts or themes using a content analysis [32]. This systematic review will follow a data based convergent qualitative synthesis to allow all studies to be analyzed using the same synthesis method for each objective and the results will be presented together. Owing to the unified nature of this type of synthesis, data transformation is required and will be accomplished by qualitative thematic analysis, therefore ‘qualitizing’ the quantitative data [33].

The process of data synthesis will be iterative in nature. To address the first objective, studies will be read to identify and examine the characteristics of the educational pathway that was undertaken by the physiotherapist and then synthesized via a clustered textual description, which intends to summarize and organize included studies into groups [34]. Popay et al 2006 endorse this method of synthesis to aid in the process of description and identifying patterns within and across the studies that have been organized into groups. The objective should inform how to cluster the included studies, and for this review, studies will be grouped according to the type of educational pathway being investigated [34].

A directed content analysis [35] will be used to address the second objective–it is deductive in nature, and as such, studies will be read with the multi-professional framework for Advanced Practice [27] in mind. All text that initially appear to represent a competency that underpins one of the four pillars of Advanced Practice will be highlighted. All highlighted passages will be coded using the predetermined codes (competencies) attributed to each pillar [27]. Any text that cannot be coded will be analyzed later to determine if it represents a new code or a subcategory of an existing code. Once all data in a study is coded, it will be sorted according to which of the four pillars it represents and connected back to the described educational pathway from which it spawned, ultimately drawing connections between the educational pathway and demonstration of the four pillars by the physiotherapist. Reading and re-reading the included studies using a comparative process against the multi-professional framework for Advanced Practice and between studies, will allow investigators to describe, organize, and interpret the data in a rigorous way [27, 33].

### Wider reflexive considerations

In the context of this review, wider reflexive considerations refer to concerns that may arise from the attempt to comprehensively synthesize relevant literature across studies of various methodologies. Firstly, academia’s partiality towards both quantitative and English publications may lend to the omittance of meaningful research of different designs or that which has been conducted in other languages. This review will attempt to maintain multivocality [45] by including all modes of primary research, including a search of the grey literature and welcoming studies published in any language–while acknowledging that the concern cannot be irradicated completely [31, 35]. Also applicable to this review are concerns regarding data integration; questions of sincerity and plausibility can arise when combining qualitative and quantitative evidence to produce synthesized review findings [33–35]. Specifically, the author’s positionality can and will influence the process of integration. To increase trustworthiness, the author(s) will use self-reflexivity to situate themselves in the research and attempt to check the influence of their preconceptions and goals in the process [45, 46]. A positionality statement from the author(s) will enable readers to interpret synthesis findings and judge trustworthiness in their own context.

### Confidence in cumulative evidence

This SMSR will use a data based convergent qualitative synthesis, which necessitates qualitizing any quantitative data prior to synthesis [33]. Consequently, the GRADE-CERQual approach will be used to assess confidence in the cumulative evidence constructed. The literature supports an approach of this nature to assess the confidence in findings from qualitative evidence syntheses. Incorporating a transparent means of evaluating confidence in the cumulative evidence may facilitate use of this evidence to inform decisions and shape policies [47].

Confidence is defined here as an assessment of the degree to which a review finding is reasonable. The CERQual approach is applied to individual systematic review findings, which are analytic conclusions describing a phenomenon or aspect of a phenomenon based on the synthesis and interpretation of the data from primary studies. The CERQual considers four categories: methodological limitations, relevance, coherence and adequacy of data. Confidence is reported in each review finding as high, moderate, low, or very low. All review findings will begin with a ‘high confidence’ rating, the assumption being that the conclusions which have been drawn are a reasonable representation of the phenomenon of interest unless proven otherwise. An iterative approach will be carried out by two reviewers, moving between the various components of the CERQual, to identify meaningful concerns that have the potential to impact the confidence in the evidence [48]. Findings are rated down by one or more levels if there are important concerns regarding any of the categories or a combination of minor concerns across multiple components.

Since one or more studies contribute data to each review finding, the CERQual assessment requires a systematic and transparent approach for detecting methodological flaws of individual studies, which will ultimately impact the confidence in a review finding. Munthe-Kaas et al. 2019 recommend that reviews use a tool at the individual assessment level that fits the review question, synthesis methods, and focuses on methodological strengths and limitations [49]. As such, the thirteen criteria of the QuADS will serve to inform the four categories of the CERQual when determining the level of confidence in each review finding. Appropriately, nine of the QuADS criteria consider methodological limitations, two consider adequacy of data and two consider relevance [40, 47, 50, 51]. The remaining CERQual component of coherence reflects overall fit between the proposed review finding and the content from the primary studies as opposed to any considerations of quality of the primary source [52].

Two reviewers will independently assess quality of evidence, disagreements will be resolved through discussion–if there is no consensus, a third reviewer will mediate. A concise, yet informative summary table of qualitative findings table will be used to summarize the confidence in the evidence for distinct review findings. A summary table is considered an integral product of a CERQual assessment; it can facilitate the use of review findings in decision making processes for stakeholders and ultimately provide consumers of the literature with the information they need to allocate suitable weight to a particular review finding [46, 48].

## Discussion

The discussion will draw connections between the knowledge gained from synthesizing the evidence describing the post-licensure educational pathways that physiotherapists are engaging in and the impact these pathways are having for the physiotherapist, particularly in relation to the demonstration of the competencies that underpin the four pillars of Advanced Practice. Review findings will be presented such that each described pathway of post-licensure education is linked to the corresponding incidence and depth of demonstration of the competencies within each pillar of Advanced Practice.

Interpreting the synthesized data in this way will provide understanding about the educational pathways that exist, and which appear to be most successful at impacting the physiotherapist. The potential impact of these research findings on the journey to establishing a standardized educational pathway to Advanced Practice for the physiotherapist include enhancing role legitimacy, facilitation of lobbying for recognition as well as integration into traditional healthcare systems [29]. The long-term implications of establishing a standardized educational pathway have the potential to improve access to care for patients, offset long wait times, and improve treatment outcomes [20–24]. Finally, this SMSR will inform future high-quality research that looks to examine the effectiveness of post-licensure educational pathways to develop the four pillars of Advanced Practice for the physiotherapist.

## Supporting information

S1 Table Completed PRISMA-P checklist. (DOCX)

S2 Table Adapted search strategies. (DOCX)
