# Supplementary material for: The educational pathway to Advanced Practice for the physiotherapist: Protocol for a systematic mixed studies review
Source: PLoS One. 2024 Sep 27;19(9):e0308921. doi: 10.1371/journal.pone.0308921 (PMC11432879; doi:10.1371/journal.pone.0308921)
Supplement: S2 Table — (DOCX) [file pone.0308921.s002.docx]

**S2 table: Adapted search strategies**

Caption: S2 table. Adapted search strategies.

**Embase**

| 1 | exp physiotherapy/ |
| --- | --- |
| 2 | exp physiotherapist/ |
| 3 | exp physiotherapy practice/ |
| 4 | "physical therap*".tw,kf. |
| 5 | "physiotherap*".tw,kf. |
| 6 | "physio-therap*".tw,kf. |
| 7 | 1 or 2 or 3 or 4 or 5 or 6 |
| 8 | exp graduate education/ |
| 9 | exp education program/ |
| 10 | exp postgraduate education/ |
| 11 | exp medical education/ |
| 12 | exp physical therapy education/ |
| 13 | exp continuing education/ |
| 14 | exp residency education/ |
| 15 | exp doctoral education/ |
| 16 | exp masters education/ |
| 17 | education/ |
| 18 | "post-licensure education".tw,kf. |
| 19 | "educational intervention".tw,kf. |
| 20 | "mentoring".tw,kf. |
| 21 | "inservice training".tw,kf. |
| 22 | "professional development".tw,kf. |
| 23 | "work experience".tw,kf. |
| 24 | "internship".tw,kf. |
| 25 | musculoskeletal manipulation/ |
| 26 | "manual therapy".tw,kf. |
| 27 | IFOMPT.tw,kf. |
| 28 | "self-directed learning".tw,kf. |
| 29 | problem based learning/ |
| 30 | "competency based education".tw,kf. |
| 31 | 8 or 9 or 10 or 11 or 12 or 13 or 14 or 15 or 16 or 17 or 18 or 19 or 20 or 21 or 22 or 23 or 24 or 25 or 26 or 27 or 28 or 29 or 30 |
| 32 | professional competence/ |
| 33 | clinical competence/ |
| 34 | exp clinical decision making/ |
| 35 | clinical reasoning/ |
| 36 | exp health care quality/ |
| 37 | exp health care delivery/ |
| 38 | exp patient satisfaction/ |
| 39 | exp treatment outcome/ |
| 40 | "patient cent* care".tw,kf. |
| 41 | "clinic* practice".tw,kf. |
| 42 | experience/ |
| 43 | ((advanced or speciali* or consultant*) adj5 (practice or practitioner*)).tw,kf. |
| 44 | (capabilit* adj3 "advanced practice").tw,kf. |
| 45 | (clinic* adj3 expert*).tw,kf. |
| 46 | ((extended or expanded) adj3 (role* or scope*)).tw,kf. |
| 47 | "skill acquisition".tw,kf. |
| 48 | health personnel attitude/ |
| 49 | professional standard/ |
| 50 | exp accreditation/ |
| 51 | career mobility/ |
| 52 | leadership/ |
| 53 | patient care/ |
| 54 | safety/ |
| 55 | risk management/ |
| 56 | cooperation/ |
| 57 | "role model".tw,kf. |
| 58 | adaptable.tw,kf. |
| 59 | innovat*.tw,kf. |
| 60 | manag*.tw,kf. |
| 61 | qualification.tw,kf. |
| 62 | exp learning/ |
| 63 | interpersonal communication/ |
| 64 | exp mentor/ |
| 65 | health educator/ |
| 66 | "knowledge translation".tw,kf. |
| 67 | self-directed learning/ |
| 68 | Research Personnel/ |
| 69 | clinical research/ or research/ |
| 70 | program evaluation/ |
| 71 | evidence based practice/ |
| 72 | "best practice".tw,kf. |
| 73 | 32 or 33 or 34 or 35 or 36 or 37 or 38 or 39 or 40 or 41 or 42 or 43 or 44 or 45 or 46 or 47 or 48 or 49 or 50 or 51 or 52 or 53 or 54 or 55 or 56 or 57 or 58 or 59 or 60 or 61 or 62 or 63 or 64 or 65 or 66 or 67 or 68 or 69 or 70 or 71 or 72 |
|  | 7 and 31 and 73 |

**CINAHL**

| S69 | S7 AND S26 AND S68 |
| --- | --- |
| S68 | S27 OR S28 OR S29 OR S30 OR S31 OR S32 OR S33 OR S34 OR S35 OR S36 OR S37 OR S38 OR S39 OR S40 OR S41 OR S42 OR S43 OR S44 OR S45 OR S46 OR S47 OR S48 OR S49 OR S50 OR S51 OR S52 OR S53 OR S54 OR S55 OR S56 OR S57 OR S58 OR S59 OR S60 OR S61 OR S62 OR S63 OR S64 OR S65 OR S66 OR S67 |
| S67 | AB "clinician scientist" |
| S66 | AB "best practice" |
| S65 | (MH "Physical Therapy Practice, Research-Based") |
| S64 | (MH "Research, Physical Therapy") |
| S63 | AB "knowledge translation" |
| S62 | (MH "Program Evaluation") |
| S61 | (MH "Health Educators") |
| S60 | AB mentors |
| S59 | (MH "Communication Skills") |
| S58 | (MH "Lifelong Learning") |
| S57 | AB qualification |
| S56 | AB adaptable |
| S55 | AB innovat* |
| S54 | (MH "Role Models") |
| S53 | (MH "Cooperative Behavior") |
| S52 | (MH "Risk Management") |
| S51 | (MH "Management") |
| S50 | (MH "Leadership") |
| S49 | (MH "Careers in Allied Health") |
| S48 | (MH "Career Mobility") |
| S47 | (MH "Accreditation") |
| S46 | (MH "Professional Role") |
| S45 | (MH "Attitude of Health Personnel") |
| S44 | (MH "Skill Acquisition") |
| S43 | AB ((advanced or speciali* or consultant*) AND (practice or practitioner*)) |
| S42 | AB ((extended or expanded) AND (role* or scope*)) |
| S41 | AB "advanced practice" AND capabilities |
| S40 | AB "clinic* practice" |
| S39 | AB "clinic* expert*" |
| S38 | (MH "Patient Centered Care") |
| S37 | (MH "Patient-Reported Outcomes") |
| S36 | (MH "Outcomes of Education") |
| S35 | (MH "Treatment Outcomes") |
| S34 | (MH "Patient Satisfaction") |
| S33 | (MH "Health Care Delivery, Integrated") |
| S32 | (MH "Quality of Health Care") |
| S31 | (MH "Clinical Reasoning") |
| S30 | (MH "Decision Making, Clinical") |
| S29 | (MH "Clinical Competence") |
| S28 | (MH "Multiskilled Health Practitioners") |
| S27 | (MH "Professional Competence") |
| S26 | S8 OR S9 OR S10 OR S11 OR S12 OR S13 OR S14 OR S15 OR S16 OR S17 OR S18 OR S19 OR S20 OR S21 OR S22 OR S23 OR S24 OR S25 |
| S25 | AB "IFOMPT" |
| S24 | (MH "Self-Directed Learning") |
| S23 | (MH "Professional Development") |
| S22 | (MH "Job Experience") |
| S21 | AB “educational intervention" |
| S20 | AB “post-licens* education” |
| S19 | AB "inservice training" |
| S18 | (MH "Problem-Based Learning") |
| S17 | (MH "Education, Competency-Based") |
| S16 | (MH "Manual Therapy") |
| S15 | (MH "Internship and Residency") |
| S14 | (MH "Clinical Supervision") |
| S13 | (MH "Mentorship") |
| S12 | (MH "Education, Physical Therapy") |
| S11 | (MH "Education, Interdisciplinary") |
| S10 | (MH "Education, Continuing") |
| S9 | (MH "Education, Masters") |
| S8 | (MH "Education, Graduate") |
| S7 | S1 OR S2 OR S3 OR S4 OR S5 OR S6 |
| S6 | AB "physical therap*" |
| S5 | AB physio-therap* |
| S4 | AB physiotherap* |
| S3 | (MH "Physical Therapy Practice") |
| S2 | (MH "Physical Therapy") |
| S1 | (MH "Physical Therapists+") |

**Web of Science**

| 1 | TS=("physical therap*") |
| --- | --- |
| 2 | TS=("physiotherap*") |
| 3 | TS=("physio-therap*") |
| 4 | #1 OR #2 OR #3 |
| 5 | TS=("graduate education") |
| 6 | TS=("post-graduate education") |
| 7 | TS=((masters) near/5 (education)) |
| 8 | TS="continuing education" |
| 9 | TS="professional education" |
| 10 | TS="post-licens* education" |
| 11 | TS="mentor*" |
| 12 | TS=residency |
| 13 | TS=fellowship |
| 14 | TS="inservice training" |
| 15 | TS="manual therapy" |
| 16 | TS="musculoskeletal manipulation*" |
| 17 | TS="job experience" |
| 18 | TS="professional development" |
| 19 | #5 OR #6 OR #7 OR #8 OR #9 OR #10 OR #11 OR #12 OR #13 OR #14 OR #15 OR #16 OR #17 OR #18 |
| 20 | TS=((advanced or speciali* or consultant* or extended or expanded) near/5 (practice or practitioner* or scope or role or physi*)) |
| 21 | TS=((professional OR clinical) AND (competence)) |
| 22 | TS=((clinical) AND (reasoning OR "decision making")) |
| 23 | TS=((quality or delivery) near/3 healthcare) |
| 24 | TS=(treatment outcome) |
| 25 | TS=(patient outcome) |
| 26 | TS=(patient satisfaction) |
| 27 | TS=(patient cent* care) |
| 28 | TS=(clinic* expert*) |
| 29 | TS=(leader*) |
| 30 | TS=(accreditation) |
| 31 | TS=(professional role) |
| 32 | TS=(role model) |
| 33 | TS=(manage*) |
| 34 | TS=qualification |
| 35 | TS=(health educator) |
| 36 | TS=(self-directed learning) |
| 37 | TS=(knowledge translation) |
| 38 | TS=communicator |
| 39 | TS=researcher |
| 40 | TS=(evidence-based practice) |
| 41 | TS=(best practice) |
| 42 | TS=(clinician scientist) |
| 43 | #42 OR #41 OR #40 OR #39 OR #38 OR #37 OR #36 OR #35 OR #34 OR #33 OR #32 OR #31 OR #30 OR #29 OR #28 OR #27 OR #26 OR #25 OR #24 OR #23 OR #22 OR #21 OR #20 |
| 44 | #43 AND #19 AND #4 |

**SportDISCUS**

| S52 | S22 OR S23 OR S24 OR S25 OR S26 OR S27 OR S28 OR S29 OR S30 OR S31 OR S32 OR S33 OR S34 OR S35 OR S36 OR S37 OR S38 OR S39 OR S40 OR S41 OR S42 OR S43 OR S44 OR S45 OR S46 OR S47 OR S48 OR S49 OR S50 OR S51 |
| --- | --- |
| S51 | AB "researcher" |
| S50 | AB "best practice" |
| S49 | AB "best practice" |
| S48 | AB "clinician scientist" |
| S47 | DE "EVIDENCE-based medicine" |
| S46 | AB "knowledge translation" |
| S45 | AB "self-directed learning" |
| S44 | AB "health educator" |
| S43 | AB "career mobility" |
| S42 | DE "ROLE models" |
| S41 | AB innovation |
| S40 | DE "DECISION making" |
| S39 | DE "CERTIFICATION" |
| S38 | DE "LEADERSHIP" OR DE "LEADERSHIP in adolescence" OR DE "LEADERSHIP scale for sports" OR DE "RECREATION leadership" OR DE "SHARED leadership" |
| S37 | AB ((advanced or speciali* or consultant*) N5 (practice or practitioner*)). |
| S36 | AB competenc* N2 “advanced practice” |
| S35 | AB capabilit* N2 “advanced practice” |
| S34 | AB "patient cent* care" |
| S33 | AB "treatment outcome" |
| S32 | AB "patient satisfaction" |
| S31 | AB "patient outcome" |
| S30 | AB "delivery of healthcare" |
| S29 | AB "quality of healthcare" |
| S28 | AB "advanced practice" |
| S27 | AB "clinical decision-making" |
| S26 | AB "clinical expert*" |
| S25 | AB "professional competence" |
| S24 | AB "clinical competence" |
| S23 | AB "clinical reasoning" |
| S22 | DE "TREATMENT effectiveness" |
| S21 | S1 OR S2 OR S3 OR S4 OR S5 OR S6 |
| S20 | S7 OR S8 OR S9 OR S10 OR S11 OR S12 OR S13 OR S14 OR S15 OR S16 OR S17 OR S18 OR S19 |
| S19 | AB "professional development" |
| S18 | AB "inservice training" |
| S17 | AB fellowship |
| S16 | AB "residency training" |
| S15 | DE "MENTORING" |
| S14 | AB "graduate education" |
| S13 | AB "post-licens* education" |
| S12 | AB "professional education" |
| S11 | AB "continuing education" |
| S10 | AB "continuing education |
| S9 | AB "master* level education" |
| S8 | AB "post-graduate education" |
| S7 | DE "EDUCATION" OR DE "ACADEMIA" OR DE "AERONAUTICS education" OR DE "COEDUCATION" OR DE "DANCE education" OR DE "HEALTH education" OR DE "OUTDOOR education" OR DE "PHYSICAL education" OR DE "PSYCHOLOGY of learning" OR DE "SEX education" OR DE "SPECIAL education" OR DE "TEACHERS" |
| S6 | (AB "physical therap*") AND (S1 OR S2 OR S3 OR S4 OR S5) |
| S5 | AB "physical therap*" |
| S4 | AB physio-therap* |
| S3 | AB physiotherap* |
| S2 | DE "PHYSICAL therapy practice" |
| S1 | DE "PHYSICAL therapy" OR DE "BALNEOLOGY" OR DE "COLD therapy" OR DE "ELECTROTHERAPEUTICS" OR DE "HYDROTHERAPY" OR DE "LIANGONG" OR DE "MANIPULATION therapy" OR DE "OCCUPATIONAL therapy" OR DE "PHOTOTHERAPY" OR DE "RECREATIONAL therapy" OR DE "SPORTS physical therapy" OR DE "THERMOTHERAPY" OR DE "VETERINARY physical therapy" |

**ProQuest Education Databases**

| **S67** | [S7] AND [S25] AND [S66] |
| --- | --- |
| **S66** | [S26] OR [S27] OR [S28] OR [S29] OR [S30] OR [S31] OR [S32] OR [S33] OR [S34] OR [S35] OR [S36] OR [S37] OR [S38] OR [S39] OR [S40] OR [S41] OR [S42] OR [S43] OR [S44] OR [S45] OR [S46] OR [S47] OR [S48] OR [S49] OR [S50] OR [S51] OR [S52] OR [S53] OR [S54] OR [S55] OR [S56] OR [S57] OR [S58] OR [S59] OR [S60] OR [S61] OR [S62] OR [S63] OR [S64] OR [S65] |
| **S65** | summary("clinician scientist") |
| **S64** | summary("best practice") |
| **S63** | MESH.EXACT("Evidence-Based Practice") |
| **S62** | MESH.EXACT("Research Personnel") |
| **S61** | summary("self-directed learning") |
| **S60** | summary("knowledge translation") |
| **S59** | MESH.EXACT("Program Evaluation") |
| **S58** | MESH.EXACT("Health Educators") |
| **S57** | MESH.EXACT("Mentors") |
| **S56** | summary(qualification) |
| **S55** | summary(manag*) |
| **S54** | [summary(innovat*)](https://www.proquest.com/recentsearches.recentsearchtabview.recentsearchesgridview.scrolledrecentsearchlist.checkdbssearchlink:rerunsearch/6A7D016AD2BE443FPQ/None/$N?t:ac=RecentSearches) |
| **S53** | summary(adaptable) |
| **S52** | summary("role model") |
| **S51** | MESH.EXACT("Cooperative Behavior") |
| **S50** | MESH.EXACT("Safety Management") |
| **S49** | [MESH.EXACT("Risk Management")](https://www.proquest.com/recentsearches.recentsearchtabview.recentsearchesgridview.scrolledrecentsearchlist.checkdbssearchlink:rerunsearch/B17E81B5E33440DCPQ/None/$N?t:ac=RecentSearches) |
| **S48** | MESH.EXACT("Patient Care Management") |
| **S47** | MESH.EXACT("Leadership") |
| **S46** | MESH.EXACT("Career Mobility") |
| **S45** | MESH.EXACT("Accreditation") |
| **S44** | MESH.EXACT("Professional Role") |
| **S43** | MESH.EXACT("Attitude of Health Personnel") |
| **S42** | summary(skill acquisition) |
| **S41** | summary(advanced practice competencies) |
| **S40** | summary(advanced practice capabilities) |
| **S39** | summary(advanced practice physiotherapy) |
| **S38** | summary(((extended or expanded) AND (scope or role))) |
| **S37** | summary(((advanced or speciali* or consultant*) AND (practice or practitioner*))) |
| **S36** | summary("clinic* experience") |
| **S35** | summary("clinic* practice") |
| **S34** | summary("clinic* expert") |
| **S33** | MESH.EXACT("Treatment Outcome") |
| **S32** | MESH.EXACT("Patient Satisfaction") |
| **S31** | MESH.EXACT("Delivery of Health Care") |
| **S30** | MESH.EXACT("Quality of Health Care") |
| **S29** | MESH.EXACT("Clinical Decision-Making") |
| **S28** | MESH.EXACT("Clinical Reasoning") |
| **S27** | MESH.EXACT("Professional Competence") |
| **S26** | MESH.EXACT("Clinical Competence") |
| **S25** | [S8] OR [S9] OR [S10] OR [S11] OR [S12] OR [S13] OR [S14] OR [S15] OR [S16] OR [S17] OR [S18] OR [S19] OR [S20] OR [S21] OR [S22] OR [S23] OR [S24] |
| **S24** | summary("IFOMPT") |
| **S23** | summary("manual therapy") |
| **S22** | summary("job experience") |
| **S21** | summary("educational intervention") |
| **S20** | summary("post-licens* education") |
| **S19** | summary("master* level education") |
| **S18** | MESH.EXACT("Problem-Based Learning") |
| **S17** | MESH.EXACT("Competency-Based Education") |
| **S16** | MESH.EXACT("Musculoskeletal Manipulations") |
| **S15** | MESH.EXACT("Internship and Residency") |
| **S14** | MESH.EXACT("Inservice Training") |
| **S13** | MESH.EXACT("Mentoring") |
| **S12** | MESH.EXACT("Preceptorship") |
| **S11** | MESH.EXACT("Education, Continuing") |
| **S10** | MESH.EXACT("Education") |
| **S9** | MESH.EXACT("Education, Professional") |
| **S8** | MESH.EXACT("Education, Graduate") |
| **S7** | [S1] OR [S2] OR [S3] OR [S4] OR [S5] OR [S6] |
| **S6** | summary("physical therap*") |
| **S5** | summary(physio-therap*) |
| **S4** | summary(physiotherap*) |
| **S3** | MESH.EXACT("Physical Therapy Modalities") |
| **S2** | MESH.EXACT("Physical Therapy Specialty") |
| **S1** | MAINSUBJECT.EXACT("Physical therapists") |

**Cochrane**

| #1 | MeSH descriptor: [Physical Therapists] explode all trees |
| --- | --- |
| #2 | MeSH descriptor: [Physical Therapy Modalities] this term only |
| #3 | MeSH descriptor: [Physical Therapy Specialty] this term only |
| #4 | (physiotherap*):ti,ab,kw (Word variations have been searched) |
| #5 | (physio-therap*):ti,ab,kw (Word variations have been searched) |
| #6 | (physical therap*):ti,ab,kw (Word variations have been searched) |
| #7 | MeSH descriptor: [Education, Graduate] explode all trees |
| #8 | MeSH descriptor: [Education, Professional] explode all trees |
| #9 | MeSH descriptor: [Interprofessional Education] explode all trees |
| #10 | MeSH descriptor: [Education, Continuing] explode all trees |
| #11 | MeSH descriptor: [Mentoring] explode all trees |
| #12 | MeSH descriptor: [Inservice Training] explode all trees |
| #13 | MeSH descriptor: [Internship and Residency] explode all trees |
| #14 | MeSH descriptor: [Musculoskeletal Manipulations] explode all trees |
| #15 | MeSH descriptor: [Problem-Based Learning] explode all trees |
| #16 | MeSH descriptor: [Competency-Based Education] explode all trees |
| #17 | MeSH descriptor: [Self-Directed Learning as Topic] explode all trees |
| #18 | (master* level):ti,ab,kw (Word variations have been searched) |
| #19 | (master* near/3 education):ti,ab,kw (Word variations have been searched) |
| #20 | (post-licens* near/3 education):ti,ab,kw (Word variations have been searched) |
| #21 | (educational intervention):ti,ab,kw (Word variations have been searched) |
| #22 | (((job or work) AND experience)):ti,ab,kw (Word variations have been searched) |
| #23 | (professional development):ti,ab,kw (Word variations have been searched) |
| #24 | (manual therapy):ti,ab,kw (Word variations have been searched) |
| #25 | (IFOMPT):ti,ab,kw (Word variations have been searched) |
| #26 | MeSH descriptor: [Professional Competence] explode all trees |
| #27 | MeSH descriptor: [Clinical Competence] explode all trees |
| #28 | MeSH descriptor: [Clinical Decision-Making] explode all trees |
| #29 | MeSH descriptor: [Clinical Reasoning] explode all trees |
| #30 | MeSH descriptor: [Quality of Health Care] explode all trees |
| #31 | MeSH descriptor: [Delivery of Health Care] explode all trees |
| #32 | MeSH descriptor: [Patient Satisfaction] explode all trees |
| #33 | MeSH descriptor: [Treatment Outcome] explode all trees |
| #34 | MeSH descriptor: [Patient-Centered Care] explode all trees |
| #35 | (clinic* near/3 expert*):ti,ab,kw (Word variations have been searched) |
| #36 | (clinic* practice):ti,ab,kw (Word variations have been searched) |
| #37 | ((capabilit* OR competenc*) near/3 (advanced practice)):ti,ab,kw (Word variations have been searched) |
| #38 | ((advanced or speciali* or consultant*) near/5 (practice or practitioner*)):ti,ab,kw (Word variations have been searched) |
| #39 | ((extended or expanded) near/3 (role* or scope*)):ti,ab,kw (Word variations have been searched) |
| #40 | (skill acquisition):ti,ab,kw (Word variations have been searched) |
| #41 | MeSH descriptor: [Attitude of Health Personnel] 1 tree(s) exploded |
| #42 | MeSH descriptor: [Professional Role] explode all trees |
| #43 | MeSH descriptor: [Accreditation] explode all trees |
| #44 | MeSH descriptor: [Career Mobility] this term only |
| #45 | MeSH descriptor: [Leadership] explode all trees |
| #46 | MeSH descriptor: [Patient Care Management] explode all trees |
| #47 | MeSH descriptor: [Safety Management] 1 tree(s) exploded |
| #48 | MeSH descriptor: [Risk Management] explode all trees |
| #49 | MeSH descriptor: [Cooperative Behavior] this term only |
| #50 | (role model):ti,ab,kw (Word variations have been searched) |
| #51 | (adaptable):ti,ab,kw (Word variations have been searched) |
| #52 | (innovat*):ti,ab,kw (Word variations have been searched) |
| #53 | (manager):ti,ab,kw (Word variations have been searched) |
| #54 | (qualification):ti,ab,kw (Word variations have been searched) |
| #55 | MeSH descriptor: [Learning] this term only |
| #56 | MeSH descriptor: [Communication] this term only |
| #57 | MeSH descriptor: [Mentors] this term only |
| #58 | MeSH descriptor: [Health Educators] explode all trees |
| #59 | MeSH descriptor: [Program Evaluation] 2 tree(s) exploded |
| #60 | (knowledge translation):ti,ab,kw (Word variations have been searched) |
| #61 | ((self-directed learning)):ti,ab,kw (Word variations have been searched) |
| #62 | MeSH descriptor: [Research Personnel] this term only |
| #63 | MeSH descriptor: [Evidence-Based Practice] explode all trees |
| #64 | (best practice):ti,ab,kw (Word variations have been searched) |
| #65 | (clinician scientist):ti,ab,kw (Word variations have been searched) |
| #66 | #1 OR #2 OR #4 OR #5 OR #6 |
| #67 | #7 OR #8 OR #9 OR #10 OR #11 OR #12 OR #13 OR #14 OR #15 OR #16 OR #17 OR #18 OR #19 OR #20 OR #21 OR #22 OR #23 OR #24 OR #25 |
| #68 | #26 OR #27 OR #28 OR #29 OR #30 OR #31 OR #32 OR #33 OR #34 OR #35 OR #36 OR #37 OR #38 OR #39 OR #40 OR #41 OR #42 OR #43 OR #44 OR #45 OR #46 OR #47 OR #48 OR #49 OR #50 OR #51 OR #52 OR #53 OR #54 OR #55 OR #56 OR #57 OR #58 OR #59 OR #60 OR #61 OR #62 OR #63 OR #64 OR #65 |
| #69 | #66 AND #67 AND #68 |
